# Internet-based relapse prevention with therapist support for help-seeking problematic alcohol users: a randomized pilot study

**DOI:** 10.1186/1940-0640-8-S1-A75

**Published:** 2013-09-04

**Authors:** Christopher Sundström, Anne H Berman, Kristina Sinadinovic, Magnus Johansson

**Affiliations:** 1 Karolinska Institute, Department of Clinical Neuroscience, Center for Psychiatric Research, Stockholm, Sweden; 2 Stockholm Center for Dependency Disorders, Stockholm, Sweden

## 

Research indicates a clear need for easily accessible interventions of different lengths and intensity for individuals with problematic substance use. Internet interventions with therapist support for problematic alcohol use have yielded positive results in prior studies, but have never been investigated in Sweden. This pilot study tested an online structured relapse prevention program with and without therapist support. 80 internet help-seekers from the general population were recruited through a national self-help site and randomized into three groups: 1) therapist support via secure messages (n=20); 2) a choice between therapist support via messages or live chats (n=20); 3) self-help (n=40). Baseline data showed no significant differences between the three groups concerning age, gender, Time Line Follow Back (TLFB), Alcohol Use Disorders Identification Test (AUDIT), Readiness to Change Questionnaire (RTCQ) and Quality of Life (WHOQOL). In the group with a choice between messages and chat, 35% chose chat. All follow-up data for the study have recently been collected, and results will be analyzed for presentation at the conference. This study has the potential to further broaden knowledge about internet-based interventions for problematic alcohol use, and provide information about the significance of therapist support.

